# Interdisciplinary Therapeutic Approaches to Atypical and Malignant Meningiomas

**DOI:** 10.3390/cancers15174251

**Published:** 2023-08-25

**Authors:** Leonidas Trakolis, Athanasios K. Petridis

**Affiliations:** Department of Neurosurgery, Agios Loukas Clinic Thessaloniki, 55236 Thessaloniki, Greece; opticdisc@aol.com

**Keywords:** aggressive meningioma, adjuvant radiotherapy, chemotherapy, Simpson classification

## Abstract

**Simple Summary:**

Recent advances in diagnostics and adjuvant therapy regarding meningiomas have called into question the oncological relevance of gross total removal. Some authors see the Simpson grading as rather obsolete and adopt a more conservative treatment. In this narrative review, we discuss the significance of the degree of resection of aggressive meningiomas in terms of oncological profit and postoperative functional outcome according to the most recent studies.

**Abstract:**

Meningiomas have the highest incidence among brain tumors. In contrast to benign tumors that constitute the majority of this tumor entity, the treatment of aggressive meningiomas (WHO Grade 2 and 3) is more challenging, requiring gross total removal of the tumor and the affected dura and adjuvant radiotherapy. Sometimes the location and/or the configuration of the tumor do not favor radical surgical resection without endangering the patient’s clinical condition after surgery and pharmacological therapy has, until now, not been proven to be a reliable alternative. Discussion: In this narrative review, we discuss the current literature with respect to the management of meningiomas, discussing the importance of the grade of resection in the overall prognosis of the patient and in the planning of adjuvant therapy. Conclusions: According to the location and size of the tumor, radical resection should be taken into consideration. In patients with aggressive meningiomas, adjuvant radiotherapy should be performed after surgery. In cases of skull base meningiomas, a maximal, though safe, resection should take place before adjuvant therapy. An interdisciplinary approach is beneficial for patients with primary or recurrent meningioma.

## 1. Introduction

Meningiomas represent more than 30% of brain tumors [1]. The prognosis varies according to the location and classification of the tumor [2,3,4]. The WHO has provided a classification according to the invasiveness of the tumor [5], while the evolution of molecular genetics has brought a newer classification (Table 1) [6,7]. The most aggressive types of meningioma are WHO Grade 2 and 3 tumors, which are related not only to more rapid growth but also to a poorer prognosis [8,9]. In MR imaging, they are usually accompanied by heterogenous contrast enhancement in T1 sequences and brain oedema in FLAIR, suggesting an aggressive type of tumor [10]. During the presentation of the WHO classification of meningiomas in 2016, WHO Grade 2 tumors were classified only as invasive meningiomas without any other signs of atypia [11], while the 2021 molecular markers were introduced as grading criteria for some subtypes [12]. Therapeutic management can vary, from radiation to gamma knife and from surgery to a conservative approach (wait and see). Systemic (pharmacological) therapy is an alternative for refractory or recurrent cases when surgery and radiation are not available, showing limited success but a promising future. As histological classification is normally available after surgery, it is difficult to plan the proper therapy in advance, which makes (aggressive) surgery the safest solution for patients. However, radical removal of the tumor is not always without sacrifices [13,14,15]. The location of the tumor [16,17] and the age of the patient [18] play major roles. The extent of resection will usually be determined before surgery according to the radiological findings and the general health condition of the patient [17,19]. In 1957, Simpson demonstrated a classification system for meningioma resection [20], dividing patients into five categories according to the type of resection (Table 2), giving the classification a prognostic role regarding recurrence. However, not everybody currently agrees with the prognostic value of the Simpson grading [8,9,17,21,22,23]. Predominantly, because of the available adjuvant radiotherapies, surgeons are increasingly opting for an incomplete resection of the tumor to prevent intraoperative complications and postoperative morbidity. In this review, we scrutinize the recent literature regarding the management of aggressive meningiomas.

## 2. Pathophysiology

Meningiomas first drew the attention of neurosurgeons in the early 1920s. Harvey Cushing named these tumors meningioma [26] and fifteen years later already had a database of 313 cases [27]. Although the exact pathophysiology is still unclear, many factors have been identified over the years that play major roles in the development of this type of tumor. The tumor owes its name to the Greek word meningia (μηνίγγια), which means dura matter. The meninges derive from two different embryological origins. Neural crest cells are responsible for telencephalic meninges, and the mesoderm is responsible for the remaining meninges [28]. Both can lead to identical tumors without any differences despite their different origins [29].

Many causes have been identified for tumorigenesis through the years. One of the best-understood pathogeneses of meningiomas is genetic background; for example, in neurofibromatosis type I and II [30]. In this case, patients suffer from multiple, usually benign, meningiomas in the whole neural system [31].

Furthermore, the modernization of our lifestyle has led to an increase in the environmental irradiation that we receive. Consequently, meningioma incidence has also increased, leading to a new subtype, i.e., radiation-induced meningiomas (RIMs) [32]. These are more common in female patients (1.33:1), and their location varies according to the site of radiation exposure. They are mostly seen in younger patients after high-dose radiation in terms of radiotherapy or after low-dose radiation exposure in patients between 50 and 60 years old [32]. The majority are associated with higher invasiveness and recurrence after surgery and radiotherapy than sporadic meningiomas [33]. According to Umansky et al. [32], these tumors are usually WHO Grade 2 and 3. However, the relationship between cell phone use, electromagnetic waves, and modern telecommunications has not been proven; this may be due to the short follow-up time [34]. Other risk factors proposed are head injury [35], smoking, alcohol [36], malnutrition, and exposure to heavy metals without any concrete clinical proof. The protective role of fresh fruits and vegetables is, however, generally accepted [37].

Some of the most discussed and complex risk factors are hormones and hormonal therapies. The higher incidence of meningiomas in the female population has pushed scientists to examine the role of estrogen, progesterone, and androgen receptors in the development of meningiomas. Further associations exist between the same hormones and also breast cancer [38]. About one-third of meningiomas express estrogen receptors, while more than two-thirds express progesterone receptors [39]. Pregnancy and hormonal therapies, e.g., oral contraceptives, have been investigated for their potential association with tumor development. However, treatment with oral contraceptives could not be linked to meningioma development [39,40,41,42,43], although there was an increase in the development of meningiomas in postmenopausal women in Sweden [44,45]. There is also an increased tumor development risk in males using androgen analogs [46]. In addition, pregnancy is clearly associated with meningioma development, probably through endocrine mechanisms [26,47]. Interestingly, treatment with tamoxifen (estrogen receptor antagonist) and antiprogestin did not produce positive and reliable results [48]. Androgen receptor antagonists have not yet been tested, and only everolimus with octreotide therapy has a satisfying effect against meningiomas [49].

## 3. Classification

**Figure 1:**
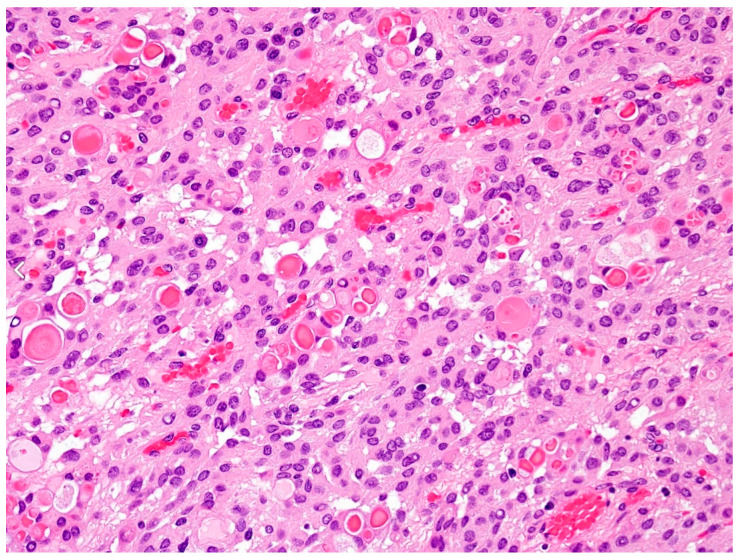
Secretory meningioma: WHO Grade 1 with eosinophilic secretions (pseudopsammoma bodies) and low mitotic rate [52]. The magnification is 100×.

**Figure 2:**
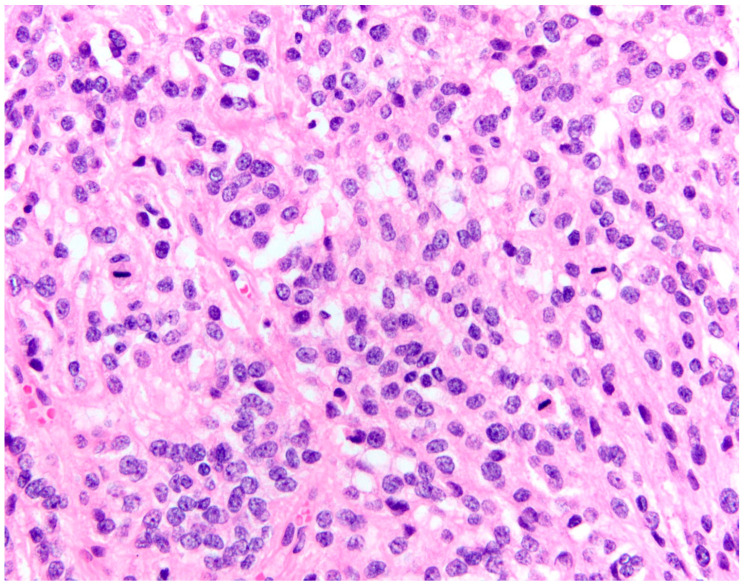
WHO Grade 3 (anaplastic) meningioma with high mitotic activity (>20 mitotic figures in 10 consecutive high-power fields) [25]. The magnification is 200×.

As already mentioned, meningiomas are classified according to their invasiveness in three major groups on the WHO scale; the most benign are WHO Grade 1 (70–80%), meaning they not only grow slower but are also not aggressive to neighboring structures (Figure 1) [50,51,52]. Regardless of their benign nature, the recurrence rate in 20 years is 20% [53]. This is probably due to the fact that no adjuvant radiotherapy takes place for Grade 1 meningiomas, and some surgeons undertake an incomplete resection to minimize the operation risks [22], especially in meningiomas of the skull base. A total of 20 to 30% of meningiomas invade brain tissue, with the majority of them being WHO Grade 2 (about 17%) and the rest being Grade 3 [24]. Meningiomas with increased mitosis, brain invasion, and/or malignant cytology [5] belong to the last two categories and are commonly called aggressive. In actively growing meningiomas, peritumoral brain edema is usually seen [10]. This is believed to be due to the high vascular permeability and increased tumor angiogenesis [54]. In 2016, the WHO revised its guidelines and criteria for meningioma classification [11]. According to this revision, there must be an unequivocal invasion of the tumor in the gray matter in order for it to be classed as Grade 2, as adherence to underlying brain tissue is not enough [11]. Brain invasion, a major marker of tumor recurrence, is a standalone criterion for a Grade 2 tumor. If the criteria for a Grade 2 meningioma are met, and the mitoses comprise more than 20 per 10 consecutive high-power fields, the meningioma is classified as Grade 3-anaplastic (Figure 2) [25].

Genetic factors may affect the recurrence rate, a fact that has led the WHO to include molecular findings in a newer classification (Table 1) [12]. The meningioma community went a step further and used molecular profiling for the prediction of recurrence [55]. Recently, it was shown that even heterozygous loss of CDKN2A/B greatly accelerates recurrence in aggressive meningioma [56]. In the last classification of the WHO in 2021, brain invasion remained an independent criterion for atypical meningioma, although some studies have questioned its prognostic relevance [57,58]. In a recent systematic review, brain invasion was associated with higher recurrence rates and shorter progression-free survival not only in atypical and malignant tumors but also in brain-invasive but otherwise benign meningiomas (BIOBMs) [59]. Nevertheless, molecular markers have been introduced as grading criteria for some subtypes, while rhabdoid and papillary meningiomas are graded on the basis of the same criteria for atypia and anaplasia as for other meningioma variants and are not only based on the subtype-specific histology. Furthermore, a CDKN2A/B homozygous deletion is associated with a worse overall outcome [60], while a TERT-promoter mutation indicates a high recurrence risk [61,62,63]. These two factors constitute independent criteria for a WHO Grade 3 meningioma regardless of any histological indications for anaplasia. Of course, such sophisticated molecular analysis is not always available and takes time after surgery, but it can still be a guide for patients to consider additional therapy after surgery. Regarding tumor recurrence rates, DNA-methylation profiling defines better the subgroups of meningiomas so it should be considered not only for recurrent tumors but also for primary tumors [64].

In line with the problem of recurrence, Simpson demonstrated in 1957 a revolutionary system for time classification depending on the extent of the tumor resection [20]. Grade I concerns macroscopically complete removal of the tumor, including its dural and bone parts, while grade V refers to simple decompression of the tumor. The Simpson classification is not only used to characterize the type of resection; it is also a strong predictor for tumor recurrence and overall survival [65]. A higher Simpson-grade resection is clearly associated with higher recurrence frequency [66]. However, this correlation is less obvious in falx, posterior fossa, and skull base meningiomas [65].

This classification soon became the holy grail of meningioma resection among neurosurgeons. Over the years, new operative technics were developed in order to achieve a Simpson Grade I resection, including in respect of skull base meningiomas, where the complexity of the anatomy is often higher [67,68]. The trend nowadays is rather conservative. There are studies showing non-progression of a tumor from its dural tail, rendering a Simpson Grade I resection obsolete in risky surgeries [69]. With the recent development of radiation therapy, there are more options than ever before to remove tumors (radiosurgery) or slow down tumor progression. Also used as adjuvant therapy after surgery, radiation therapy increasingly dominates clinicians’ choices. From gamma knife radiosurgery [70] to fractionated external beam [71] and single-fraction stereotactic radiation [72], the results are positive for small benign and/or unresectable tumors.

### 3.1. Malignant vs. Benign Meningiomas

Aggressive meningiomas require a more aggressive approach [73]. However, it is almost impossible to preoperatively distinguish aggressive from non-aggressive meningiomas. There are some clues in the preoperative imaging that suggest the tumor’s aggressiveness. Magnetic resonance imaging (MRI) is the gold standard for the diagnosis of brain tumors. In the case of meningiomas, a well-circumscribed, dural-based, and mostly homogenously enhancing lesion presents. Some of these come with a so-called dural tail, while others have different characteristics, like a cystic core or calcifications [74]. Necrosis and oedema in FLAIR are common signs of malignity and aggressiveness, although peritumoral oedema is not rare in secretory (WHO Grade 1) meningiomas [24].

Thus, it is difficult to know with certainty before surgery whether or not the meningioma is aggressive. Modern approaches try to differentiate between benign and anaplastic meningiomas through more sophisticated diagnostic methods [51,75,76]. However, they remain expensive and inaccessible for most hospitals. Another aspect to consider is the infiltration of a venous sinus (for example, the sagittal sinus in parafalcine meningiomas) or involvement of the cerebral vessels and/or cranial nerves, commonly seen in skull base meningiomas with the encasement of different cranial nerves, the basilar or the carotid arteries and/or their branches. Such tumors inevitably have a worse prognosis, as a radical resection is usually impossible. They may also be classified as WHO Grade 1 but have a poorer prognosis [77] and higher recurrence rates [78].

### 3.2. Meningiomatosis

One of the most complicated situations is the presence of multiple meningiomas in one patient. In meningiomatosis, there are multiple tumors that are mostly unihemispheric and rarely in the posterior fossa; there is also a female predominance. Less than 10% of patients with meningiomas have multiple tumors, and more than half of these cases are associated with neurofibromatosis type 2 [79]. The other most common causes of meningiomatosis are hormones and radiation [80,81]. The fact that the pathophysiology of meningiomatosis is still unclear makes it more difficult to handle the condition. Understanding the genetics of this condition is even more important than in solitary tumors. There are two dominant theories about the pathophysiology of meningiomatosis: the monoclonal and the multicentric. According to the first theory, clonal migration through the cerebrospinal fluid is suggested [82,83], while chromosome 22 deletion or inactivation of the same chromosome X strengthens the monoclonal cell theory [84,85,86,87]. However, there are also some studies indicating a multicentric origin [88,89].

One-third of cases have meningiomas of a different nature [90], and it is advised to approach every tumor individually and in an interdisciplinary manner [91]. However, peritumoral brain oedema, rapid growth, and/or mass effects are red flags indicating surgical removal [92]. TRAF 7 mutation was associated in an adolescent with meningiomatosis treated with Everolimus and Bevacizumab [93], while a more extensive skull base and falx meningiomatosis completely disappeared after gamma knife radiosurgery [94]. Three cases of female patients with meningiomatosis were successfully treated with the antiprogesterone receptor agent mifepristone (RU486) [95]. Not every meningiomatosis case has a mild course, however. There are numerous cases reported with distal metastasis of meningiomas [96,97,98]. The incidence of malignant metastatic meningiomas is less than 1 per 1000, but they are related to a particularly poor prognosis [99]. A radical resection followed by adjuvant radiotherapy seems to be beneficial [99,100].

## 4. Therapeutical Options

Not every diagnosed meningioma has to or can be resected. The age and physical condition of patients play major roles in treatment decisions [101]. In addition, the progress of the tumor is an important clue for the nature of the tumor and should be followed. In cases where the tumor is small (under 2.5 cm), without neurological deficits, and with calcified parts [102], or is not easily accessible, a follow-up examination is usually considered. Long-term follow-ups are mostly for small or inaccessible tumors; the alternative for small or inaccessible tumors is radiation therapy. During the last two decades, radiotherapy has become the first option for small lesions or skull base meningiomas encasing important vessels [103]. Recent instances of fractionated and hypofractionated radiosurgery have shown some astonishing results in tumor control (85–100% over 5 years) [104,105].

### 4.1. Surgery

Surgery still remains the therapy of choice in most cases. In recent years, tremendous evolution of surgical techniques has been achieved (neuronavigation, tractography, endoscopic methods, etc.). Surgeries have become faster and more accurate, with incisions smaller and complications rarer [106]. Under this prism, one would expect even more clinicians to strive for radical resection. However, radical removal is not always possible. The location of the tumor or the encasement of important vessels and nerves can sometimes lead to partial resection to avoid neurological deterioration of the patient. Furthermore, the age and clinical condition of the patient should be taken into consideration when planning long and/or complex surgery. Aggressive meningiomas (Grade 2 and 3) have high recurrence rates over five years of about 50–55% and 70–78%, respectively. Conversely, Grade 1 meningiomas have rates of only 7–23% [24]. However, these high percentages concern patients who undergo gross total resection. In cases of subtotal resection, the recurrence rates are much higher, depending on the Simpson grade of resection [24,65]. In such cases, adjuvant treatment is recommended, especially when tumor growth is suspected in follow-up examinations.

If recurrence is suspected, the patients should be treated with extra care. As most of the patients with Grade 3, as well as some with Grade 2 meningiomas, will undergo adjuvant radiotherapy after surgery, radiation possibilities in case of recurrence are exhausted [107]. Furthermore, radiation-induced brain changes after adjuvant radiotherapy are not rare and sometimes difficult to differentiate from recurrent tumors [64]. Thankfully, recent developments in neuroimaging and the expression of somatostatin receptor 2 in meningiomas help us to differentiate between healthy brain tissue, postoperative changes, and recurrent tumors [108]. DOTATATE and DOTATOC PET imaging are valuable diagnostic means that are nowadays widely used for this purpose [109,110,111]. Moreover, DOTATATE PET imaging has special importance in the diagnosis of intraosseous meningiomas and should be used, if possible, for improved detection of the tumor [112]. In case of confirmed recurrence, surgery is still the state-of-the-art management and should always be taken into consideration, if possible [64,113,114,115,116]. Lemée et al. reported a longer time-to-retreatment for patients with recurrent meningiomas which were surgically removed, than for those treated with radiation therapy, although the benefits of a surgical treatment of meningioma recurrences decrease with the number of surgeries [116]. However, the higher complication rates and neurological deterioration of the re-operated patients should be taken into consideration when deciding the right treatment for recurrent meningiomas [114,115]. If re-surgery is not possible, targeted radiosurgery, radiation (in cases without former adjuvant therapy), or re-radiation can be considered [64,107,117].

A review of the literature on the surgical results of anaplastic meningiomas identified the following outcomes: Rogers et al. [118] reported on 53 patients with total and subtotal resection of WHO 3 meningiomas, stating that the 3-year progression-free survival (PFS) was 58.8% and the overall survival (mean follow up: 4.8 years) was 78.6%. All patients received radiation therapy after resection. In WHO 2 meningiomas, the 3-year PFS was 93.8% in N = 52 patients who received surgery and radiation [119]. Another study evaluated 214 patients with WHO 2 meningiomas with a follow-up time of 53.4 months. The authors reported that patients who had a gross total resection (n = 158) had a significantly longer PFS and overall survival than patients with subtotal resection (n = 56) [120]. A fourth study analyzed 23 patients with WHO 2 and 3 meningiomas with total and subtotal excision and postsurgical radiotherapy. The authors showed PFS values of 58% for WHO 2 and 20% for WHO 3. The overall survival rates for WHO 2 and 3 were 83% and 23%, respectively [121]. In a study by Weber et al., the authors reported their results for 78 patients with WHO 2 meningiomas and resection of Simpson Grade I-III with postsurgical radiation, showing a 3-year PFS of 88.3% and a 3-year survival of 98.2% [122].

### 4.2. Adjuvant (Radio)Therapy

Adjuvant radiotherapy is not only important for meningiomas belonging to Grade 2 and 3 categories, as many suppose. With recurrence rates of up to 23% in spite of total resection for Grade 1 meningiomas, possible remnants should be treated [123]. Concerning aggressive meningiomas, an increased tendency for adjuvant therapy has been reported [124]. This is particularly important as recent studies show an increasing mortality and morbidity rate in these cases [125]. The type of radiation used is individually decided according to the location, size, and grade of the tumor. Fractionated external-beam radiotherapy is now a useful tool for skull base meningiomas [126] and partially resected tumors [127]. Proton and photon radiotherapy are also good options that have been shown to be beneficial when combined [128]. Adjuvant radiotherapy is also important for incompletely removed meningiomas, as it controls remnants against recurrence and improves overall survival and progression-free survival rates. A Grade 2 meningioma after Simpson I/II resection still has a recurrence rate of 30–40%, while a Grade 3 meningioma after Simpson I/II resection has a 50–80% rate over 5 years [107]. The progression-free survival rate in Grade 3 meningiomas doubles after adjuvant radiotherapy from 28 to 57% [100]. Similar results are seen in Grade 2 meningiomas, although the rates are controversial [129]. A meta-analysis in patients with atypical meningioma showed better progression-free and overall survival rates in patients who received postoperative radiotherapy, regardless of whether there was gross total or subtotal resection [130]. In patients with anaplastic meningiomas, adjuvant therapy showed improved survival rates, although there was no significant advantage in the overall survival rate [131].

However, radiation therapy is not harmless. Larger tumors need more radiation, which can reach up to 70 Gy [107,123]. This leads to complication rates of up to 23% [132], including malignant transformation and healthy brain toxicity [133,134]. Smaller lesions respond better to hypofractionated radiation therapy, and the complication rates are decreased [132]. Radiosurgery has become a good alternative therapy to surgery for small/inaccessible tumors or patients who cannot undergo surgery, such as elderly patients or patients with poor general health. Notably, in tumors with diameters under 3 cm, gamma knife therapy can achieve local control comparable to a Simpson Grade I resection [135]. Recently, two prospective non-randomized trials suggested the effectivity of radiotherapy in WHO Grade 2 meningiomas, supporting its role in progression-free survival [119,122]. However, the outcome in WHO Grade 3 meningiomas is poor due to the propensity of these tumors to invade other tissues (brain, bone) and the risk of normal tissue toxicity [118]. Further studies about the impact of radiotherapy are relatively scarce for high-grade meningiomas. An extensive review of the current evidence and future applications of radiotherapy in meningioma treatment has been provided by Chen et al. [134].

### 4.3. Pharmacological Therapy

The development of pharmacological therapy against aggressive and recurrent meningiomas started many years ago. These agents are considered against refractory and/or invasive meningiomas when surgery and radiation have failed or are not possible or available [48,136]. Cytotoxic agents, like hydroxyurea, were thoroughly tested, and although they were well-tolerated, they had limited efficacy [48,136,137,138]. Hormone therapies were also intensively tested, showing rather disappointing results [136]. This has led to a need for more targeted therapy [139]. A variety of targeted therapies like tyrosine kinase inhibitors [139,140] and everolimus monotherapy [141,142], or in combination with octreotide [49,143], have shown promising results.

Immunotherapy with interferon-α has been effective against low-grade recurrent meningiomas [144,145], but in recent years, no further studies have confirmed these results. However, some newer studies have shown promising results; understanding the underlying tumorigenesis genetics and block has been utilized in modern immunotherapy to develop effective checkpoint inhibitors. Meningiomas are known to exhibit several immune checkpoint proteins. PD-L1 expression is one of the most important, not only being proportional to tumor grade but also predicting a poorer overall prognosis due to higher recurrence and tumor progression rates [146,147,148]. A Phase 2 study of pembrolizumab, a PD-1 inhibitor, delivered encouraging efficacy in patients with recurrent and residual high-grade meningiomas [149]. Further checkpoint proteins that are highly expressed in meningiomas are PD-L2, B7-H3, CTLA-4, and NY-ESO, but their role in future therapy is still under investigation [150,151,152].

Currently, more effort is being given to the development of new pharmacological therapies. In order to achieve this, many studies have concentrated on decoding the mechanism of brain invasion. As meningiomas are highly vascularized tumors, there is special interest in inhibiting angiogenesis. Several antiangiogenic agents have also been tested, mostly in patients with recurrent tumors. They were, in most cases, well tolerated; however, the efficacy of these agents, which were partially combined with chemotherapy, has still to be proven in prospective studies [153,154,155]. Canstatin, an angiogenesis inhibitor, was expressed more than twice as frequently in non-invasive meningiomas than in invasive ones [156]. Another group proposed bevacizumab, an antiangiogenic agent, as the most effective therapeutic agent [157]; however, we should note that the cohort was small, with only 23 patients [158]. Bevacizumab as a monotherapy, or in combination with everolimus and sunitinib, are currently the most promising systemic agents for patients with aggressive meningiomas where radiation has failed [159]. Furthermore, a recent study confirmed the role of CDKN2A mRNA expression as a biomarker of clinically aggressive meningiomas, especially in Grade 3 meningiomas. This could be of great help for the development of therapeutic implications [160]. Last but not least, the future could lie in gene therapy. The idea of using adenoviruses and herpes simplex virus to insert genetic material (DNA or RNA) into human cells in order to correct or compensate for a gene abnormality or defect is not new [161,162]. However, the uncontrollable insertion of mutations and the short period of therapeutic effects have led to fewer preclinical studies and more confined development than expected [48].

Some therapies that are available in the USA and are FDA-approved were scrutinized by Jungwirth et al. [163], showing very promising results for some agents like ixabepilone, a cytotoxic agent widely used in advanced and metastatic breast cancer [164,165,166]. Alpelisib, a phosphoinositide 3-kinase α (Pi3Kα) specific inhibitor, is under investigation in combination with the MEK inhibitor trametinib (NCT03631953). Especially for patients with NF 2 mutations, a phase II trial of focal adhesion kinase inhibition (GSK2256098) showed promising efficacy and met the PFS6 criteria for further evaluation [167]. Due to the novel targets identified in recent years, there are many ongoing trials of systemic therapies for progressive and/or recurrent meningiomas [140,168,169]. A complete list of current pharmacotherapy targets can be found in the systematic review of Shahbandi et al. [168].

## 5. Discussion

While most practitioners agree that surgery is the first therapeutical option for (rapidly) progressing meningiomas, how radical the surgery should be is still a matter of debate [17]. Ideally, gross total resection followed by adjuvant radiotherapy should be the standard therapy for aggressive meningiomas [59,100,170]. Lesions located in the skull base and the posterior fossa are notorious for postsurgical complications [171,172]; the most common complications are CSF leakage and cranial nerve and vascular injuries, which are independent of the tumor grade but have been associated with a lower Simpson Grade resection. Schneider et al. [171,172] reported high complication rates in Simpson Grade I resections of skull base meningiomas. Similar resection limitations were experienced by Giammatei et al. [173] with clinoidal meningiomas. Simpson Grade I resection was achieved in about 64% of patients, showing again that the tumor location and its accessibility play crucial roles in the resection grade.

The problem of recurrence in incomplete resected meningiomas is not new. In 1957, Simpson et al. [20] stressed the role of radical resection, characterizing tumor recurrence as a “failure in surgery”, suggesting a failure to identify and remove the infiltrated tissues. Such a “failure” is sometimes inevitable, and the resection should be as radical as possible within the limits of a safe surgical procedure. After a systematic review of 26 studies, Giordan et al. [174] were confronted with the same dilemma, this time in meningiomas involving the superior sagittal sinus, i.e., either radical resection leading to morbidity or a higher risk of recurrence. They came to the conclusion that although radical (aggressive) resection has lower recurrence rates, it is associated with a higher complication rate and greater morbidity. Due to the increased prevalence of Grade 1 meningiomas, many surgeons have reevaluated the efficacy of aggressive surgery with adjuvant radiotherapy. A study analyzing the Japanese National Meningioma Databank showed a surgical complication rate of more than 19% [175], while radiosurgery boasted lower rates of up to 15.9% (mostly below 10%, according to other studies) [176].

Considering the low recurrence rates of Grade 1 meningiomas, it is sometimes understandable that surgeons are not willing to risk an aggressive resection in tumors that are difficult to access. Radical tumor resection in one study did not show any benefits in the 5-year recurrence/progression-free survival compared to more conservative resection [22]. As Oya [175] reported, there is no standard risk-to-benefit ratio for aggressive resections that allows neurosurgeons to make a (correct) decision about the therapy. However, a Simpson Grade IV resection showed a significantly higher recurrence rate [177]. Unfortunately, aggressive meningiomas are identified mostly after surgery. As a result, 20–30% of aggressive meningiomas are undertreated if aggressive surgery does not take place.

It is a fact that the location of the tumor [65] and the experience of the surgeon play important roles in surgical outcomes but not necessarily in recurrence rates. Complex surgeries in difficult-to-access regions require expertise to reach a lower Simpson Grade resection and lower complication rates [73,178]. Subtotal resection in patients with WHO Grade 2 and skull base meningiomas seems to predict a worse outcome [179]. Luckily, skull base meningiomas are mostly Grade 1 (about 95%) [179], so even if an aggressive resection is not possible, the overall survival rates remain high [179] also in respect of Simpson Grade IV and V resections [180]. This means that in cases of vessel or nerve encasement or other subjective difficulties during surgery, subtotal resection can be considered. However, it is still important to leave as little of the tumor behind as possible in favor of the success of adjuvant therapy [77] and the overall prognosis [181].

In convexity and parafalcine meningiomas, gross total removal is usually possible. With the use of the modern techniques of preoperative evaluation (CT-phlebography, MR-tractography, transcranial magnetic stimulation, etc.) and intraoperative monitoring (electrophysiology, awake brain surgery), lesions in difficult locations (central region), or with sagittal sinus infiltration can nowadays be more radically removed. Due to the fact that, in most cases, the neurosurgeon is not aware of the grade of the tumor until the histological report arrives (usually some days after surgery), there is generally a more aggressive attitude towards accessible tumors [65].

Recent studies have shown a clear advantage of adjuvant radiotherapy in patients with aggressive meningiomas, especially for WHO Grade 3 cases, improving not only the overall survival but also the progression-free survival [130,131,134,182,183]. In addition, gross total resection is associated with increased progression-free survival, although Chun et al. saw no improvement in the overall survival in atypical meningiomas [183]. Nevertheless, there are cases where radiation is not very effective. NOTCH3+ cells are found to be expressed throughout high-grade meningiomas that are resistant to radiotherapy [184].

**Figure 3:**
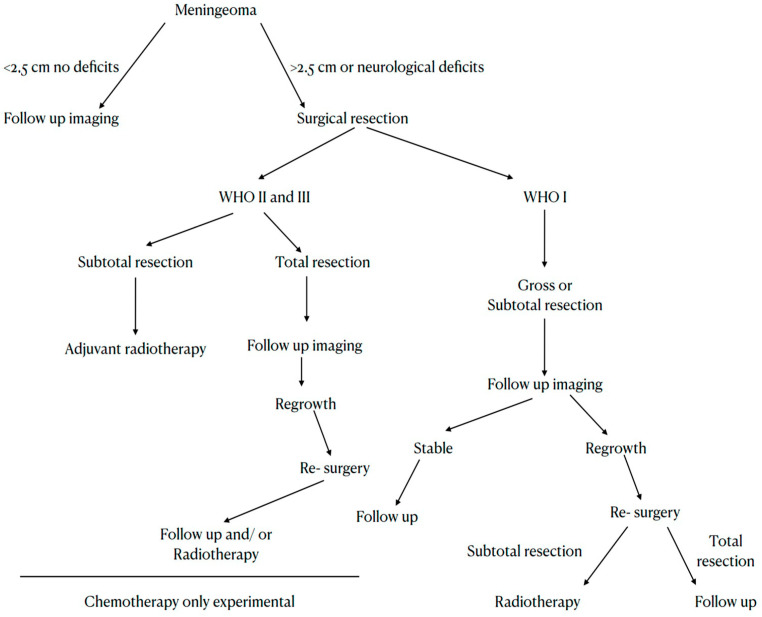
Flowchart of the currently recommended meningioma therapy.

Two years ago, the European Association of Neuro-Oncology (EANO) updated its recommendations for the diagnosis and treatment of meningiomas [185]. In aggressive meningiomas, which means tumors that radiologically look like meningiomas and show rapid growth, surgery is the first step in making a diagnosis and removing mass effects. Surgeons should strive for a Simpson Grade I resection. In WHO Grade 2 meningiomas, regular follow-ups after surgery (every six months, the first 5 years, and then annually) are highly recommended. Postsurgical radiation is indicated in cases where only a Simpson Grade IV or V resection was achieved. For WHO Grade 3 meningiomas, fractionated radiotherapy should take place after surgical removal of the tumor. The resection has to be as radical as possible because these tumors show early recurrence, rapid growth, risk of systemic metastasis, and particular molecular features at genetic and epigenetic levels. For both WHO Grade 2 and 3, there is no established systemic therapy until now [185]. Currently, surgery is the state-of-the-art management for meningioma recurrence since none of the pharmacological therapeutic alternatives show promising results in progression-free and overall survival [64,113,114,115,116]. Nevertheless, an interdisciplinary approach to each of these cases is vital for the patient, especially for more complicated cases of recurrent, multiple, or very aggressive meningiomas. Radiosurgery and radiotherapy have rapidly developed in recent years, offering a reliable option not only for small tumors but also for multiple, recurrent, or aggressive meningiomas, either as adjuvant therapy or as monotherapy. A flowchart of the currently recommended therapy is provided in Figure 3.

## 6. Conclusions

In summary, meningiomas are mostly benign tumors, but they should be approached with extraordinary care. They can occur either on the skull base or at the convexity and falx. Tumor location is important for surgery, as some tumors are more accessible than others. However, it is generally accepted that an aggressive resection should be undertaken as long as this remains safe for the patient. As we cannot pre- or perioperatively predict the WHO Grade of the tumor, it is of vital importance to remove most of the tumor, and the neighboring infiltrated tissues in order to increase the patient’s overall survival rate and morbidity. Of similar importance is adjuvant therapy for Grade 2 and 3 meningiomas, as well as regular follow-up examinations. However, Grade 1 tumors sometimes tend to recur, so follow-up examinations are recommended for all patients after meningioma resection. Systemic therapy is developing, but not as quickly as expected. Nevertheless, immunotherapy and gene therapy may be the future for the treatment of recurrent and/or refractory meningiomas. Simpson grading is, in our opinion, still up to date concerning aggressive meningiomas, as it is an excellent prognosing factor of recurrence. However, complex or inaccessible meningiomas of the skull base with vessel or nerve encasement should be removed as radically as possible without endangering the postoperative condition of the patient. Finally, large referral centers usually have more experience with poorly accessible or infiltrative tumors and possess more technical possibilities by which to remove such tumors.

## Figures and Tables

**Table 1:** Meningioma grading criteria [11,24,25].

WHO Grade	Description
Grade 1	Low mitotic rate, <4 per 10 HPFs No brain invasion 9 histologic subtypes
Grade 2 (atypical-aggressive)	Mitotic rate 4–19 per 10 HPF or Brain invasion or ≥3 or 5 specific histologies: • Spontaneous or geographic necrosis; • Patternless sheet-like growth; • Prominent nucleoli; • High cellularity; • Small cells with high n:c ratio.
Grade 3 (anaplastic-aggressive and malignant)	Mitotic rate > 20 per 10 HPF (HPF: high-power field) or Specific histologies: papillary or rhabdoid (since WHO 2021 graded on the basis of the same criteria for atypia and anaplasia as for other meningioma variants). Since WHO 2021: frank anaplasia (melanoma-, sarcoma- or carcinoma-like histology) CDKN2A and/or CDKN2B homozygous deletion TERT promoter mutation

**Table 2:** Simpson grading of meningioma resection [20].

Grade 1	Macroscopically complete tumor removal with affected dura and underlined bone.
Grade 2	Macroscopically complete tumor removal with coagulation of affected dura only.
Grade 3	Macroscopically complete tumor removal without removal of affected dura or underlying bone
Grade 4	Subtotal tumor resection
Grade 5	Tumor decompression with or without biopsy
